# The complete chloroplast genome of an inverted-repeat-lacking species, *Vicia sepium*, and its phylogeny

**DOI:** 10.1080/23802359.2018.1431071

**Published:** 2018-01-24

**Authors:** Chaoyang Li, Yunlin Zhao, Huimin Huang, Yi Ding, Yinhui Hu, Zhenggang Xu

**Affiliations:** a Hunan Research Center of Engineering Technology for Utilization of Environmental and Resources Plant, Central South University of Forestry and Technology, Changsha, Hunan, P.R. China;; b School of Material and Chemical Engineering, Hunan City University, Yiyang, Hunan, P.R. China

**Keywords:** Chloroplast genome, inverted-repeat-lacking, phylogeny, *Vicia sepium*

## Abstract

*Vicia sepium* L., as a pan-temperate species, it is suitable as a constituent part of grazed grasslands because it can be resistant to winter kill and it contains abundant proteins. In this study, complete chloroplast genome of *V. sepium* was explored and phylogenetic analysis was done. The result showed that the whole genome was 124,095 bp long with IR loss. The overall GC content was 35.03%. A total of 109 genes were identified, including 80 protein-coding, 30 transfer RNA, and four ribosome RNA genes. Similar to most of the IRLC plastoms, *rpl22*, *rps16* and one intron of *clpP* were lost. The phylogenetic analysis of protein-coding genes from 25 Fabaceae species belonging to IRLC showed that *V. sepium* was similar to *V. sativa.* The chloroplast genome reported here will promote our understanding of the phylogeny and evolution of the genus *Vicia*.

*Vicia sepium* L., in subfamily Papilionoideae of Fabaceae, is a well-known species widely distributed in Europe, Central Asia, and North Asia, introduced from the Old World to North America. As a pan-temperate species, it is suitable as a constituent part of grazed grasslands because it can be resistant to winter kill and it contains abundant proteins (Maršalkienė 2016). However, the taxonomic history of *Vicia* L. is confused and contentious which has led misapplication of names of *V. sepium*. Compared to other angiosperms, the inverted-repeat-lacking clade (IRLC) including *V. sepium* exists diversity of changes in structural rearrangements of chloroplast genome (Sabir et al. 2014). Then, there are disagreements among authors concerning the classification of *Vicia* L. because of its high morphological variability (Shiran and Raina 2001; Iamonico et al. 2017). Therefore, we sequenced the complete chloroplast genome of *V. sepium* to enhance our understanding of its evolution processes and breeding.

The mature leaves of *V. sepium* were obtained from a natural population in Dongting Lake region (28°48′46.06″N, 112°21′10.19″E) under liquid N_2_ and stored at −80 °C with accession number 20170707JJ. The total chloroplast DNA was extracted with Plant Chloroplast Purification Kit (BTN120308) and Column Plant DNA Extraction Kit and sequenced by using Illumina Hiseq 4000 Platform. Filtration and annotation of the whole genome and drawing of a circular map referred to Zhang’s method (Zhang et al. 2017) by using Trimmomatic v0.32, DOGMA and cpGAVAS, OGDRAW. The complete chloroplast genome was submitted to the NCBI database under the accession number MG682352.

The complete chloroplast genome of *V. sepium* was 124,095 bp long with IR loss. The overall GC content was 35.03%. A total of 109 genes were identified, including 80 protein-coding, 30 transfer RNA, and four ribosome RNA genes. A total of 12 genes (*ndhA*, *rrn23S*, *ndhB*, *ycf2*, *rpl2*, *accD*, *atpF*, *ropC1*, *rpoB*, *clpP*, *ycf3*, *psaA*) contained introns: *ycf3* contained three introns, *ycf2* contained two introns and the rest of the genes contained a single intron. Similar to most of the IRLC plastoms, *rpl22*, *rps16* and one intron of *clpP* were lost. Phylogenetic relationships were presented using 54 conserved chloroplast protein sequences shared among 24 published Fabaceae species and *V. sepium* by using the maximum likelihood method with 1000 bootstrap replicates based on Muscle progress in MEGA7.0 (Figure 1) (Kumar et al. 2016). Our phylogenetic analysis confirmed the relationship between *V. sepium* and *V. sativa* within the Fabaceae. The chloroplast genome reported here will promote our understanding of the phylogeny and evolution of the genus *Vicia*.

**Figure 1.**
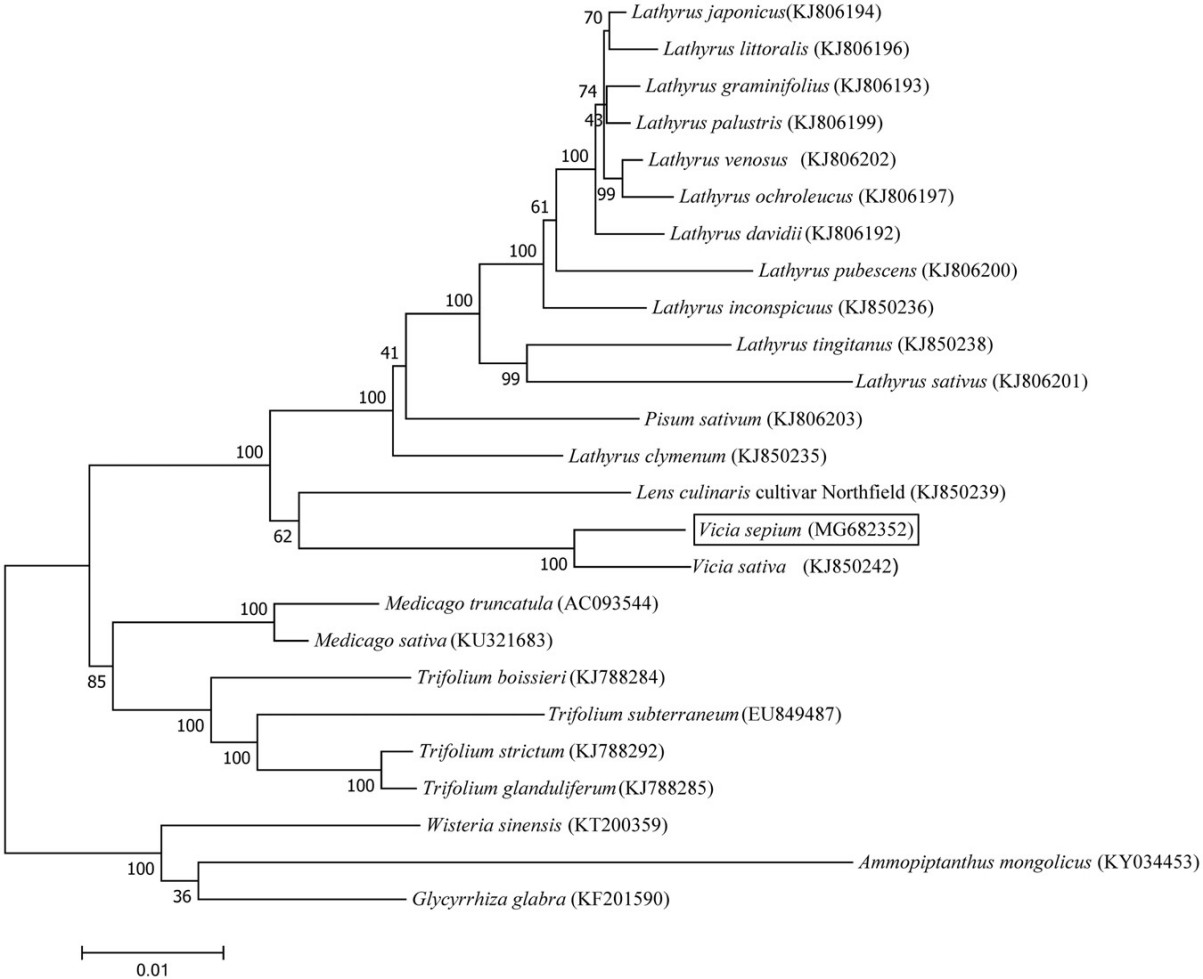
Maximum likelihood phylogenetic tree of *V. sepium* with 24 species in Fabaceae family.
